# Supplementary material for: Optimizing and Implementing a Community-Based Group Fall Prevention Program: A Mixed Methods Study
Source: Int J Environ Res Public Health. 2024 Jan 31;21(2):162. doi: 10.3390/ijerph21020162 (PMC10887802; doi:10.3390/ijerph21020162)
Supplement: Supplementary file 1 [file ijerph-21-00162-s001.zip › ijerph-2755999-supplementary.pdf]

Supplementary materials

Appendix S1. Evaluation In Balance program

Introduction

We are curious about your experiences with the In Balance program. That is why this questionnaire is about your experience and opinion about the Balance program.

Instruction

Answer the following questions by putting a cross next to the answer that suits you best. Completing the questionnaire takes a maximum of 15 minutes.

The following questions are about the In Balance program.

1. **Have you attended all the meetings of the In Balance program?**

- ○ Yes, continue to question 2
- ○ No, continue to question 1a and 1b

1a. If you did not attend all the meetings of the In Balance program, how many meetings have you missed?

____

1b. If you did not attend all the meetings of the In Balance program, what was the main reason for this (you may tick several boxes)?

- ○ Following the In Balance program took me too much time
- ○ Due to health problems
- ○ I could not bring myself to do it
- ○ I had other activities
- ○ I had problems getting to the location
- ○ Because of COVID-19
- ○ Due to another reason, namely:

____

2. **Did COVID-19 play a role in your choice to participate in the In Balance study (you can tick several boxes)?**

- ○ Yes, I wanted more physical and social activity again
- ○ Yes, I doubted about group activities and measurements
- ○ Yes, for another reason, namely ______________________________
- ○ No

3. **Have you done the exercises at home?**

- ◯ Yes, proceed to question 3a
- ◯ No, continue to question 3b

3a. If you have done exercises at home, how often have you done the exercises?

- ◯ Multiple times a week
- ◯ Once per week
- ◯ Less than once per week

Continue to question 4

3b. If you did not do any exercises at home, what was the main reason for this (you can tick several boxes)?

- ◯ Doing the exercises at home took me too much time
- ◯ Because of health problems
- ◯ I could not bring myself to do it
- ◯ I didn't have a suitable room in which I could do the exercises
- ◯ I found the exercises too difficult/dangerous
- ◯ I found the exercises too tiring

4. **How satisfied are you with the total In Balance program you followed?**

- ◯ Very satisfied
- ◯ A little satisfied
- ◯ Not satisfied, but also not dissatisfied
- ◯ A little dissatisfied
- ◯ Very dissatisfied

5. **How satisfied are you with the course meetings (the course meetings are the first 4 meetings) of the In Balance program that you followed?**

- ◯ Very satisfied
- ◯ A little satisfied
- ◯ Not satisfied, but also not dissatisfied
- ◯ A little dissatisfied
- ◯ Very dissatisfied

6. **How satisfied are you with the training sessions (the training sessions you have done in the last 10 weeks) of the In Balance program that you followed?**

- ○ Very satisfied
- ○ A little satisfied
- ○ Not satisfied, but also not dissatisfied
- ○ A little dissatisfied
- ○ Very dissatisfied

7. **What do you think of the length of time of each meeting?**

- ○ Too short
- ○ Just right
- ○ Too long

8. **What do you think of the number of meetings?**

- ○ Too little
- ○ Just right
- ○ Too much

9. **What do you think of the time between 2 meetings?**

- ○ Too short
- ○ Just right
- ○ Too long

10. **What do you think of the duration of the total In Balance program?**

- ○ Too short
- ○ Just right
- ○ Too long

11. **Suppose someone you know has trouble with balance or walking, would you recommend this person the In Balance program?**

- ○ Yes
- ○ Maybe
- ○ No

12. **Suppose you have (more) trouble with your balance in the future, would you follow the In Balance program again?**

- ○ Yes
- ○ Maybe
- ○ No

13. **What did you think of the contact with the other participants?**
- ◯ I liked the contact with other participants
- ◯ I didn't like the contact with other participants, but I didn't find it unpleasant either
- ◯ I did not like the contact with the other participants

14. **What did you think of the group size?**
- ◯ I thought the group was too big
- ◯ I thought the group was just right
- ◯ I thought the group was too small

<u>The following questions are about your health.</u>

15. **Do you feel that you are less likely to get tired after following the In Balance program?**
- ◯ Yes, I get tired less quickly
- ◯ Yes, I get tired a little less quickly
- ◯ I get tired just as quickly
- ◯ No, I get tired a little faster
- ◯ No, I get tired much faster

16. **Do you feel that your balance has been improved after following the In Balance program?**
- ◯ Yes, my balance improved a lot
- ◯ Yes, my balance has improved slightly
- ◯ My balance has remained the same
- ◯ No, my balance has deteriorated slightly
- ◯ No, my balance has deteriorated a lot

17. **Do you feel that you have become physically stronger after following the In Balance program?**
- ◯ Yes, I have become much stronger
- ◯ Yes, I have become a little stronger
- ◯ I remained equally strong
- ◯ No, I have become a little less strong
- ◯ No, my strength deteriorated a lot

18. **Have you gained more confidence in yourself while doing your daily activities?**
- ◯ Yes, I gained much more confidence
- ◯ Yes, I gained a little more confidence
- ◯ My confidence remained the same
- ◯ No, I am a little less confident
- ◯ No, I am much less confident

The following questions are about your Balanced therapist.

19. **How satisfied are you with the teacher who gave the In Balance program?**
- ○ Very satisfied
- ○ A little satisfied
- ○ Not satisfied, but also not dissatisfied
- ○ A little dissatisfied
- ○ Very dissatisfied

20. **Was the teacher capable to tailor the In Balance program for you if necessary?**
- ○ The therapist was certainly able to tailor the In Balance program
- ○ The therapist was a bit able to tailor the In Balance program
- ○ The therapist was not able to tailor the In Balance program

The next question is about costs.

21. **If a personal contribution was requested for your participation in the 14-week In Balance program, how much would you be willing to pay?**
- ○ I would not want to pay a personal contribution
- ○ 1 to 25 euros
- ○ 25 to 50 euros
- ○ 50 to 75 euros
- ○ 75 to 100 euros
- ○ More than 100 euros

Closing

22. **Would you like to add something about the In Balance program? We'd love to hear from you. Below you have space to write this down.**

____

____

____
